# Hyper-radiosensitivity affects low-dose acute myeloid leukemia incidence in a mathematical model

**DOI:** 10.1007/s00411-022-00981-7

**Published:** 2022-07-21

**Authors:** Sjors Stouten, Ben Balkenende, Lars Roobol, Sjoerd Verduyn Lunel, Christophe Badie, Fieke Dekkers

**Affiliations:** 1 Center for Environmental Safety and Security, National Institute for Public Health and the Environment (RIVM), Bilthoven, The Netherlands; 2 Department of Mathematics, Utrecht University, Utrecht, The Netherlands; 3 Cancer Mechanisms and Biomarkers group, Radiation Effects Department, Radiation, Chemical and Environmental Hazards, UK Health Security Agency, Chilton, Didcot, Oxon, OX11 0RQ UK

**Keywords:** Low dose, Acute myeloid leukemia, CBA mice, Mathematical modeling, Ionizing radiation exposure, Hyper-radiosensitivity

## Abstract

In vitro experiments show that the cells possibly responsible for radiation-induced acute myeloid leukemia (rAML) exhibit low-dose hyper-radiosensitivity (HRS). In these cells, HRS is responsible for excess cell killing at low doses. Besides the endpoint of cell killing, HRS has also been shown to stimulate the low-dose formation of chromosomal aberrations such as deletions. Although HRS has been investigated extensively, little is known about the possible effect of HRS on low-dose cancer risk. In CBA mice, rAML can largely be explained in terms of a radiation-induced Sfpi1 deletion and a point mutation in the remaining Sfpi1 gene copy. The aim of this paper is to present and quantify possible mechanisms through which HRS may influence low-dose rAML incidence in CBA mice. To accomplish this, a mechanistic rAML CBA mouse model was developed to study HRS-dependent AML onset after low-dose photon irradiation. The rAML incidence was computed under the assumptions that target cells: (1) do not exhibit HRS; (2) HRS only stimulates cell killing; or (3) HRS stimulates cell killing and the formation of the Sfpi1 deletion. In absence of HRS (control), the rAML dose-response curve can be approximated with a linear-quadratic function of the absorbed dose. Compared to the control, the assumption that HRS stimulates cell killing lowered the rAML incidence, whereas increased incidence was observed at low doses if HRS additionally stimulates the induction of the Sfpi1 deletion. In conclusion, cellular HRS affects the number of surviving pre-leukemic cells with an Sfpi1 deletion which, depending on the HRS assumption, directly translates to a lower/higher probability of developing rAML. Low-dose HRS may affect cancer risk in general by altering the probability that certain mutations occur/persist.

## Introduction

One of the early observations among atomic bomb survivors in Hiroshima and Nagasaki was an increased risk of developing leukemia (Folley et al. 1952). Since then, many epidemiological analyses have been presented on the incidence of various forms of leukemia in the life span study cohort of Japanese atomic bomb survivors to investigate, among others, the shape of the dose-response curve (Preston et al. 1994; Richardson et al. 2009; Hsu et al. 2013). In these analyses, excess risk models with a linear, linear-quadratic or a purely quadratic dependency in radiation dose are typically fitted to cohort data to examine the possible form of the dose-response curve that best describes the data. Another approach is to translate the (limited) radiobiological understanding of a disease into a mechanistic mathematical model to study the dose-response curve (Preston 2017; Shuryak 2019; Kaiser et al. 2021).

Stouten et al. (2021) presented a mathematical model to quantify the dose-response curve of the major radiation-induced acute myeloid leukemia (rAML) pathway in photon-irradiated male CBA/H mice. These mice have been used extensively to study rAML due to very low background incidence, reproducible maximum rAML induction of about 20% following 3 Gy of whole-body exposure, and histopathological features similar to human AML (Major and Mole 1978; Mole et al. 1983; Verbiest et al. 2015). The major murine rAML disease pathway can be explained in terms of two mutations affecting gene Sfpi1 coding for hematopoietic transcription factor PU.1 (Finnon et al. 2012; Verbiest et al. 2015; O’Brien et al. 2020). A radiation-induced deletion with Sfpi1 copy loss is the first hit responsible for the formation of pre-leukemic cells (Bouffler et al. 1997; Silver et al. 1999), and is identified in about 82% of the rAML cases. In approximately 78% of these rAML cases, the cells with an Sfpi1 deletion additionally acquired a specific point mutation in the remaining Sfpi1 allele (O’Brien et al. 2020). These two mutations are considered to be responsible for the formation of leukemic cells and the resulting rAML onset (Cook et al. 2004; Suraweera et al. 2005; Verbiest et al. 2018).

Although the target cells responsible for rAML development remain unknown, hematopoietic stem and progenitor cells (HSPCs) are generally thought to be involved in leukemogenesis (Passegué et al. 2003; Hope et al. 2004; Taussig et al. 2005; Hirouchi et al. 2011; Shlush et al. 2014; Gault et al. 2019). Recent in vitro clonogenic survival experiments revealed that murine HSPCs such as long-term hematopoietic stem cells (LT-HSCs) exhibit cellular hyper-radiosensitivity (HRS) (Rodrigues-Moreira et al. 2017). Low-dose HRS is responsible for severely lowering the surviving cell fraction after very low dose exposure compared to what one would expect based on a linear-quadratic cell survival model. Further increasing the dose activates an increased radioresistance mechanism, which causes the surviving cell fraction to increase again. At higher doses, the surviving cell fraction converges back onto the traditional linear-quadratic model (Marples and Collis 2008). In the present paper, the term HRS includes the response in the entire dose region where clonogenic cell survival is lower than expected based on a linear-quadratic model, i.e., it includes the increased radioresistance phenomenon. Rodrigues-Moreira et al. (2017) observed that the potential target cells of rAML display HRS for acute doses below 0.1 Gy, with a maximum effect around 0.06 Gy. A transient low-dose radiation-induced increase of reactive oxygen species has been shown to be responsible for inducing HRS in these hematopoietic cells (Rodrigues-Moreira et al. 2017).

Cellular HRS has been extensively investigated for the endpoint of cell survival (Lambin et al. 1993; Short et al. 1999; Joiner et al. 2001; Marples and Collis 2008; Olobatuyi et al. 2018); however, it remains extraordinarily difficult to relate the possible effects of HRS to the endpoint of cancer risk. Besides the endpoint of cell survival, HRS has also been shown to stimulate the induction of radiation-induced chromosomal aberrations and deletions at very low doses (Seth et al. 2014; Troshina et al. 2020). The rAML incidence may be affected if HRS stimulates the formation of the Sfpi1 deletion at very low doses.

Exposure to doses typically absorbed during diagnostic procedures such as PET/CT scans may affect risk estimates if target cells exhibit HRS. Because of a small effect size at lower doses, it is not realistic/practical to conduct mouse experiments to infer a dose-response curve dependent on the possible HRS status of rAML target cells. In the present study, a contribution is made to expand the (scarcely) available literature about the possible effects of HRS on the low-dose rAML incidence. The induced-repair model introduced by Marples and Joiner (1993) was used to investigate the effect of HRS target cell status on the low-dose rAML incidence. In the present paper, three scenarios are considered to study how cellular HRS may influence the incidence of low-dose rAML. The assumptions were made that HRS does not affect cell survival (HRS$^-$), HRS only influences cell survival (HRS$^+_1$), or HRS stimulates cell killing (i.e., it influences cell survival) as well as the induction of the Sfpi1 deletion (HRS$^+_2$). Based on the presented model, experiments are proposed to possibly detect whether HRS affects low-dose rAML incidence. Furthermore, the computationally intensive stochastic rAML model from Stouten et al. (2021) was redesigned such that the dose-response curve can be calculated almost instantaneously.

## Materials and methods

### Background of the model

**Fig. 1:**
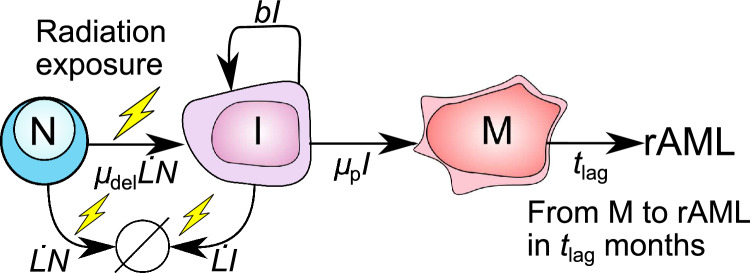
Overview of the two-mutation rAML model. Normal murine bone marrow cells (*N*) are assumed to transform into pre-leukemic cells *I* due to a radiation-induced deletion with Sfpi1 copy loss. Intermediate cells *I* proliferate and can transform into malignant cells *M* due to the occurrence of a point mutation in the remaining Sfpi1 allele. Both cells *N* and *I* can additionally undergo radiation-induced cell death (*N*, *I* $\rightarrow \varnothing$). Once a mouse acquires a single malignant cell, the time required for rAML onset and diagnosis is *t*_lag_ months, and only occurs if the mouse survives sufficiently long. With the exception of latency *t*_lag_, the rates along the arrows correspond to the transition rates included in the differential equations for *N*, *I* and *M* (Eqs. 3–5). The effect of hyper-radiosensitivity (HRS) on leukemogenesis was studied with the assumptions that HRS only affects the per cell death rate ($\dot{L}$) of cells *N* and *I* or that HRS stimulates cell killing as well as the formation of the Sfpi1 deletion (*N* $\rightarrow$ *I*). The HRS assumptions were incorporated into the model by replacing the rate $\dot{L}$ with the HRS-dependent rate $\dot{L}_\text {HRS}$

The redeveloped rAML model presented here including an HRS extension is based on previous modeling work (Dekkers et al. 2011; Stouten et al. 2021) in which, similar to the two-stage models for cancer risk assessment, malignant cells are formed due to the occurrence of two mutations (Moolgavkar et al. 1988; Dewanji et al. 1989; Leenhouts and Chadwick 1994). Figure 1 shows an overview of the model utilized to quantify the rAML incidence. Briefly, the mathematical CBA/H mouse rAML model assumes that normal HSCs (*N*) are transformed into pre-leukemic intermediate cells (*I*) due to a radiation-induced interstitial deletion on chromosome 2 with Sfpi1 copy loss. Cells *N* and *I* can both die due to radiation exposure. Cells *I* proliferate and they transform into malignant cells when the codon R235 point mutation occurs in the remaining Sfpi1 allele. The formation of the first malignant cell leads to rAML onset over the course of $t_\text {lag}$ months, provided that the mouse does not die during that time (Dekkers et al. 2011; Stouten et al. 2021). The expressions along the arrows correspond with the rates (except for latency *t*_lag_) used in the differential equation model to describe the response of bone marrow cells (*N*, *I* and *M*) to ionizing radiation exposure.

The mathematical male CBA/H mouse model developed by Stouten et al. (2021) enables one to determine the distribution of potential rAML diagnosis times ($f_\text {A}(t)$). Here, an essential observation is that, if mice did not die from other causes, every mouse would eventually develop rAML. This allows one to define, for each mouse, two independent time points: $t_\text {A}$, the potential time at which rAML occurs in the absence of other causes of death, and $t_{\overline{\text {A}}}$, the potential time at which a mouse dies in the absence of rAML. The potential rAML diagnosis time ($t_\text {A}$) is obtained by adding the diagnosis time latency ($t_\text {lag}$) to the time at which the first malignant cell is formed ($t_{M=1}$). Thus, the rAML diagnosis can only take place if a mouse survives sufficiently long to develop rAML, i.e., $t_{M=1}+t_\text {lag}=t_\text {A} \le t_{\overline{\text {A}}}$. Similar to Stouten et al. (2021), the addition of a diagnosis latency of $t_\text {lag}$ = 5.06 months was based on the observation that mice, which were deleted of exon 5 of the Sfpi1 gene (PU.1^−/−^), developed AML with a median latency of 22 weeks (Metcalf et al. 2006). This latency estimate is almost identical to a model-based estimation made by Dekkers et al. (2011).

In the present paper, the computationally intensive stochastic rAML model developed by Stouten et al. (2021) is replaced by a more efficient model. Instead of running time-consuming simulations to check whether rAML could have been diagnosed per mouse ($t_\text {A} \le t_{\overline{\text {A}}}$), a differential equation model is used to directly determine the probability of rAML development. The probability distribution of the potential time at which the first malignant cell is formed, $f_{M=1}(t)$, can be derived from the differential equation model. The potential rAML diagnosis distribution time, $f_\text {A}(t)$, in absence of any other causes of death can be found from $f_{M=1}(t)$ with the aforementioned time lag $t_\text {lag}$. Furthermore, the dose-dependent probability distribution of the potential time to non-rAML causes of death, $f_{\overline{\text {A}}}(t)$, is known (Stouten et al. 2021). By assuming that $t_\text {A}$ and $t_{\overline{\text {A}}}$ are independent, one can utilize the distributions $f_{\overline{\text {A}}}(t)$ and $f_\text {A}(t)$ to find the distribution of the actual rAML diagnosis time:

1 $$\begin{aligned} f_\text {d}(t)= \bigg (1-\hat{F}_{\overline{\text {A}}}(t)\bigg )f_\text {A}(t), \end{aligned}$$

where $\hat{F}_{\overline{\text {A}}}(t)$ is the (corrected) cumulative distribution function of $f_{\overline{\text {A}}}(t)$ which will be defined later. At time *t*, $1-\hat{F}_{\overline{\text {A}}}(t)$ represents the probability that a mouse has not yet died from non-rAML causes. The probability of developing rAML is found by calculating the area under the curve of $f_\text {d}(t)$, i.e., ${\mathbb {P}}(\text {rAML})={\mathbb {P}}(t_\text {A} < t_{\overline{\text {A}}})=\int _{0}^{\infty }f_\text {d}(t)\mathrm{d}t$. The distribution $f_\text {d}(t)$ is called improper because $0\le \int _{0}^{\infty } f_\text {d}(t)\mathrm{d}t<1$.

### Differential equation model of bone marrow leukemogenesis

By translating the two-mutation model of rAML (Dekkers et al. 2011; Verbiest et al. 2015; Stouten et al. 2021) into differential equations, the potential rAML diagnosis time distribution ($f_\text {A}(t)$) can be obtained and used to find the actual rAML diagnosis time distribution ($f_\text {d}(t)$).

The lethal event/lesion formation rate $\dot{L}(t)$ was derived from a dose-dependent linear-quadratic model ($L(D)=\alpha D +\beta D^2$), and can be used to model the clonogenic cell survival fraction *S* following exposure to a dose *D* (Gy) through: $S(D)=\exp (-L(D))$ (Chadwick and Leenhouts 1973; Kellerer and Rossi 1974). The rate $\dot{L}(t)$ can be utilized to describe the radiation-induced loss of clonogenic potential in differential equations and is obtained from *L*(*D*) after substitution of the dose absorption function $D(t)=\dot{D}t$: $L(t)=\alpha \dot{D}t + \beta \dot{D}^2t^2$, where $\dot{D}$ is the constant dose rate (Gy/month) with which mice are irradiated. Taking the time derivative of *L*(*t*) yields the rate $\dot{L}(t)$ (Zaider and Minerbo 2000; Gong et al. 2013; Olobatuyi et al. 2018):

2 $$\begin{aligned} \dot{L}(t) = {\left\{ \begin{array}{ll} \alpha \dot{D} + 2 \beta \dot{D}^2t &{} \text {if } 0\le t \le T=D/\dot{D}\\ 0 &{} \text {otherwise}. \end{array}\right. } \end{aligned}$$

Note that, irradiation starts at time $t=0$, the dose of interest *D* accumulates at exposure time $T=D/\dot{D}$ and $L(t=T)=L(D)$.

The following ordinary differential equations describe the dynamics of the number of normal (*N*), intermediate (*I*) and malignant (*M*) bone marrow cells in *absence* of HRS (note: Fig. 1 can be used as a reference for how each model rate corresponds to a certain process):

3 $$\begin{aligned} \dot{N}(t)&= -\dot{L}(t)N(t) -\mu _\text {del}\dot{L}(t)N(t), \end{aligned}$$

4 $$\begin{aligned} \dot{I}(t)&= \mu _\text {del}\dot{L}(t)N(t) + I(t)(b -\mu _\text {p} -\dot{L}(t)), \end{aligned}$$

5 $$\begin{aligned} \dot{M}(t)&= \mu _\text {p}I(t), \end{aligned}$$

where the parameters $\mu _\text {del}$ (dimensionless), *b* (month^−1^) and $\mu _\text {p}$ (month^−1^) correspond to the formation of the Sfpi1 deletion, the proliferation rate and the Sfpi1 point mutation rate respectively. The assumption was made that the number of bone marrow cells with a deleted Sfpi1 copy can be described through a linear-quadratic model *L*(*D*) (Stouten et al. 2021). This assumption is based on the observation that the number of lethal events and chromosome aberrations are linearly correlated (McMahon 2018), and the increase in the number of interstitial deletions is a linear-quadratic function of the radiation dose (Cornforth et al. 2002). Note that no distinction was made between the radiation-induced cell killing rate ($\dot{L}$) of cells *N* and *I*, because cells *I* only differ from cells *N* in the occurrence of the radiation-induced Sfpi1 deletion. Hence, the assumption was made that, during the very brief exposure time, the occurrence of the Sfpi1 deletion does not alter the radiosensitivity of cells *I*. It was additionally assumed that normal cells *N* could not transition into *I* due to a naturally occurring interstitial Sfpi1 deletion, and proliferation of *N* was excluded because acute exposure was considered here ($T\approx 0$). It should further be noted that the model developed by Stouten et al. (2021) contains an additional intermediate cell compartment in which Sfpi1-deleted cells do not have a growth advantage in the early stages after irradiation (Olme et al. 2013a). This compartment is ignored in the presented model because it requires an extra parameter and it makes the model solution more complex, while yielding similar results.

Assuming that malignant cell formation/arrival from intermediate cells follows a Poisson process with rate function $\dot{M}(t)$, then the time required to produce the first malignant cell after irradiation has the following probability distribution (Hurtado and Kirosingh 2019):

6 $$\begin{aligned} f_{M=1}(t)=\dot{M}(t)e^{-M(t)}. \end{aligned}$$

The potential rAML diagnosis time distribution in the absence of other causes of death is obtained by shifting the curve $f_{M=1}(t)$ with $t_\text {lag}$ months:

7 $$\begin{aligned} f_\text {A}(t) = {\left\{ \begin{array}{ll} 0 &{} \text {if } t< t_\text {lag}\\ f_{M=1}(t-t_\text {lag}) &{} \text {otherwise}. \end{array}\right. } \end{aligned}$$

The equations for $\dot{N}$, $\dot{I}$ and $\dot{M}$ (Eqs. 3–5) need to be solved to use the above density function to quantify the rAML incidence. Stouten et al. (2021) derived a dose-dependent expression for the number of intermediate cells present at time $T\approx 0$ following brief high-dose-rate exposure ($I_0(D)$), by assuming that no cells *I* proliferate or transform into *M* during exposure. By reproducing this approach with initial conditions $N(0)=N_0\approx$15,670 (Staber et al. 2013; Stouten et al. 2021) and $I(0)=0$, the following initial condition can be found:

8 $$\begin{aligned} I_0(D) = N_0 e^{-L(D)(1+\mu _\text {del})} \Big (e^{\mu _\text {del}L(D)} -1 \Big ). \end{aligned}$$

The equations $\dot{I}(t)=(b-\mu _\text {p})I(t)$ and $\dot{M}(t)=\mu _\text {p} I(t)$ *after* radiation exposure can now easily be solved with the initial conditions $I(0)=I_0(D)$ and $M(0)=0$:

9 $$\begin{aligned} I(t)&= I_0(D)e^{(b-\mu _\text {p})t}, \end{aligned}$$

10 $$\begin{aligned} M(t)&= \frac{\mu _\text {p}}{b-\mu _\text {p}}\Big (I(t)- I_0(D)\Big ). \end{aligned}$$

The above expressions are required to use $f_\text {A}(t)$ (Eq. 7).

### Incorporation of hyper-radiosensitivity

Hyper-radiosensitivity was included in the model due to the observation that the possible target cells of rAML exhibit HRS (Rodrigues-Moreira et al. 2017). Besides the cell survival endpoint, HRS has also been observed for the endpoint of chromosomal aberrations in gamma-irradiated human peripheral G_2_ blood lymphocytes (Seth et al. 2014). Furthermore, the induced-repair model from Marples and Joiner (1993) can be utilized to describe HRS for the endpoints of cell survival, chromosomal aberrations and deletions in B14-150 Chinese hamster cells irradiated with carbon ions (Troshina et al. 2020). Based on these experimental findings, the following three scenarios were considered to study the possible effect of HRS on the rAML incidence. First, the HRS$^-$ assumption presumes that bone marrow cells do not exhibit HRS (control scenario). The second scenario assumes that bone marrow cells exhibit HRS and this only affects cell survival (HRS$^+_1$). The third scenario assumes that HRS affects cell survival and stimulates the formation of the Sfpi1 deletion (HRS$^+_2$).

The lethal event rate was modified in accordance with the induced-repair model from Marples and Joiner (1993) to describe low-dose HRS through the $\alpha$ parameter of the linear-quadratic model:

11 $$\begin{aligned} \alpha (D) = \alpha _\text {r} \Bigg (1+\Big (\frac{\alpha _\text {s}}{\alpha _\text {r}}-1 \Big )e^{-\frac{D}{D_\text {c}}}\Bigg ), \end{aligned}$$

where $\alpha _\text {r}$ represents the traditional linear-quadratic parameter $\alpha$ applied to the conventional high-dose response, $\alpha _\text {s}$ is the slope at a very low radiation dose, and $D_\text {c}$ reflects the dose at which the induction of increased radioresistance is 63% complete. The induced-repair cell survival model is given by $S_\text {HRS}(D)=e^{-\alpha (D)D - \beta D^2}$ (Marples and Joiner 1993).

The rate function, $\dot{L}_\text {HRS}(t)= \alpha (D)\dot{D}+2\beta \dot{D}^2t$, was used to describe clonogenic cell death for the HRS$^+$ assumptions *during* exposure. Rate $\dot{L}_\text {HRS}(t)$ was substituted for $\dot{L}(t)$ in the differential equations for *N* (Eq. 3) and *I* (Eq. 4) to model the possible effect of HRS on the development of rAML. For the HRS$_1^+$ scenario, only the cell death rate during irradiation was modified (i.e., $\dot{N} = -\dot{L}_\text {HRS}N -\mu _\text {del}\dot{L}N; \, \dot{I} = -\dot{L}_\text {HRS}I +\mu _\text {del}\dot{L}N$), whereas the death rate and the Sfpi1-induction rate were both changed for the HRS$_2^+$ scenario (i.e., $\dot{N} = -\dot{L}_\text {HRS}N(1+\mu _\text {del}); \, \dot{I} = \dot{L}_\text {HRS}(\mu _\text {del}N-I)$).

Again, by assuming that no cells *I* proliferate or transform into *M* during the brief exposure time, one can easily solve the differential equations for the HRS$^+$ assumptions. This yields the initial condition for the number of cells with an Sfpi1 deletion present after irradiation for the HRS$^+$ assumptions:

12 $$\begin{aligned} I_{0,\text {HRS}_1^+}(D)&= N_0 e^{-\big (\mu _\text {del}L(D) + L_\text {HRS}(D) \big )} \Big (e^{\mu _\text {del}L(D)} -1 \Big ), \end{aligned}$$

13 $$\begin{aligned} I_{0,\text {HRS}_2^+}(D)&= N_0 e^{-\big (\mu _\text {del}\hat{L}_\text {HRS}(D) + L_\text {HRS}(D) \big )} \Big (e^{\mu _\text {del}\hat{L}_\text {HRS}(D)} -1 \Big ). \end{aligned}$$

Substitution of the above initial conditions for $I_0(D)$ in Eqs.  (9) and (10) allows one to quantify the HRS-dependent rAML incidence. The $\alpha _\text {s}/\alpha _\text {r}$-ratio is unknown for the possible HRS-mediated low-dose induction of Sfpi1 loss. The dose-response curve of different types of chromosomal aberrations observed in gamma-irradiated human G_2_ blood lymphocytes display $\alpha _\text {s}/\alpha _\text {r}$ ratios of 2.5 and 3.5 (Seth et al. 2014). For simplicity, the relationship $\alpha _\text {s}=3\alpha _\text {r}$ was assumed. This ratio is utilized in the $\hat{L}_\text {HRS}(D)$ function shown in Eq. (13) and only affects the induction of the Sfpi1 deletion.

### Deaths from non-rAML causes

The dose-dependent survival time distribution ($f_{\overline{\text {A}}}(t)$) of male CBA/H mice (Major and Mole 1978) in the *absence* of rAML was approximated with a skew normal distribution with location, scale and shape parameters of $\xi$ = 25.86−0.57*D* months, $\omega$ = 5.87 months and $\alpha =$ −  1.01 respectively (Stouten et al. 2021). The distribution parameters were fixed in accordance with the observation that the mean male CBA/H mouse survival time decreases from 22.5 to 19.1 months, when the dose is increased from 0 to 6 Gy, with a survival time standard deviation of 4.83 months and a skewness of approximately − 0.141 (Major 1979). To exclude negative distribution times from the skew normal distribution, the cumulative distribution function $F_{\overline{\text {A}}}(t)$ corresponding to the density $f_{\overline{\text {A}}}(t)$ was corrected:

14 $$\begin{aligned} \hat{F}_{\overline{\text {A}}}(t)= \frac{F_{\overline{\text {A}}}(t)-F_{\overline{\text {A}}}(0)}{1-F_{\overline{\text {A}}}(0)}. \end{aligned}$$

Thus, the corrected cumulative distribution function, $\hat{F}_{\overline{\text {A}}}(t)$, was defined such that it has the properties $\hat{F}_{\overline{\text {A}}}(0)=0$ and $\hat{F}_{\overline{\text {A}}}(t)\rightarrow 1$ as $t\rightarrow \infty$. The function $\hat{F}_{\overline{\text {A}}}(t)$ was included in Eq. (1) to find the actual rAML diagnosis time distribution.

### Model implementation, data and fitting procedure

The model was implemented in R version 4.0.3 (R Core Team 2018) and a nonlinear least-squares fitting procedure (R package minpack.lm) was performed to minimize the differences between the model and the data. Values for the parameters *b* and $\mu _\text {p}$ were determined by fitting the model to dose-dependent (0.75, 1.5, 3.0, 4.5 and 6.0 Gy) rAML incidence percentages (Major 1979; Mole et al. 1983), and time-dependent cumulative rAML incidence percentages observed after 4.5 Gy of exposure (Mole et al. 1983). These data were obtained from experiments in which male CBA/H mice were irradiated with high-dose-rate X-rays. Specifically, the following cost function that is dependent on parameter vector ${\mathbf {p}}=(b, \, \mu _\text {p})$ was minimized:

15 $$\begin{aligned} C({\mathbf {p}}) =&\sum _{i=1}^{20} \bigg (w_1(i)\Big ( y(i)-\hat{y}(i,{\mathbf {p}})\Big ) \bigg )^2 \nonumber \\&\quad +\sum _{i=1}^{20} \Bigg ( w_2 \Bigg ( \frac{z(t_i)}{z(t_{20})}- \frac{\hat{z}(t_i,{\mathbf {p}})}{\hat{z}(t_{20},{\mathbf {p}})} \Bigg ) \Bigg )^2. \end{aligned}$$

The first term of the cost function takes 20 observed (*y*(*i*)) and modeled ($\hat{y}(i,{\mathbf {p}})$) dose-dependent incidence percentages into account ( $i \in \{1, \ldots , 20\}$). Each rAML incidence model-data residual for point *i* was weighted by the fraction of mice used to acquire data point *i*: $w_1 (i)=n_{\text {mice},i}/\sum _{j}n_{\text {mice},j}$.

The second term of the cost function describes the differences between observed ($z(t_i)$) and modeled ($\hat{z}(t_i,{\mathbf {p}})$) rAML incidence after acute 4.5 Gy of exposure as a function of time at time points $t_i$ ( $i \in \{1,\ldots , 20\}$). The time-dependent cumulative rAML incidence values were normalized relative to the final time point $t_{20}$, $z(t_i)/z(t_{20})$ and $\hat{z}(t_i)/\hat{z}(t_{20})$, and weighted by the mean of the rAML incidence data observed after acute 4.5 Gy of exposure ($w_2$) (Major 1979; Mole et al. 1983). This correction was applied such that both the model and the experimental data reached an identical maximum value at time $t_{20}$.

**Table 1:** Model parameter values used in this work reported with (if applicable) their model fit start values and standard errors

Parameter	Unit	Start	Value
$\alpha _\text {r}$	$\hbox {Gy}^{-1}$	–	0.0402$^\mathrm{a}$
$\alpha _\text {s}$	$\hbox {Gy}^{-1}$	–	20$^\mathrm{b}$
$D_\text {c}$	$\hbox {Gy}^{1}$	–	0.060$^\mathrm{c}$
$\beta$	$\hbox {Gy}^{-2}$	–	0.122$^\mathrm{a}$
$\mu _\text {del}$	–	–	0.0498$^\mathrm{a}$
$t_\text {lag}$	Months	–	5.06$^\mathrm{a}$
*b*	$\hbox {Month}^{-1}$	0.0624$^{\rm a}$	0.0995 ± 0.00376
$\mu _\text {p}$	$\hbox {Month}^{-1}$	$6.87 \times 10^{{-5}^{\rm a}}$	$2.17 \times 10^{-5} \pm 2.12 \times 10^{-6}$

$^\mathrm{a}$Values taken from Stouten et al. (2021)

$$^\mathrm{b}$$Assumed value

$^\mathrm{c}$Value taken from Rodrigues-Moreira et al. (2017)

Stouten et al. (2021) showed that $\alpha$ = $\alpha _\text {r}$ = 0.0402 Gy^−1^, $\beta$ = 0.122 Gy^−2^ and $\mu _\text {del}$ = 0.0499 (dimensionless) can be used to describe cell survival curves of murine HSCs and HSPCs (Mohrin et al. 2010) and approximate the relative in vitro/vivo formation of the Sfpi1 deletion in CBA/H mice following 3 Gy of X-ray exposure (Olme et al. 2013a). For the current paper, it was not possible to obtain significant parameter values when fitting all of the model parameters to the rAML incidence data at once. Lack of inclusion of cell survival data and Sfpi1 deletion data in the fitting procedure made it impossible to identify unique optimal parameter values. Hence the parameter values for $\alpha$, $\beta$ and $\mu _\text {del}$ were taken from Stouten et al. (2021) because those values can be related to experimental data. The (fitted) model parameters to run the simulations are reported in Table 1.

Rodrigues-Moreira et al. (2017) observed that the surviving fraction curve of LT-HSCs displays low-dose HRS. This HRS effect reached a maximum after about 0.06 Gy of exposure, with a corresponding surviving fraction of about 0.65. To simulate this effect, the parameter values of $D_\text {c}=0.06$ Gy and $\alpha _\text {s}=20$ Gy^-1^ were assumed.

## Results

### Low-dose HRS affects pre-leukemic cell formation

The presented mathematical model can be used to study the possible effects of HRS on the incidence of rAML in male CBA/H mice after acute high-dose-rate X-ray exposure. Simulations were carried out with the assumptions that (1) bone marrow cells do not exhibit HRS (HRS$^-$), (2) cells display HRS for the endpoint of cell survival only (HRS$_1^+$), (3) or HRS affects cell survival *and* stimulates the formation of the Sfpi1 deletion (HRS$_2^+$).

**Fig. 2:**
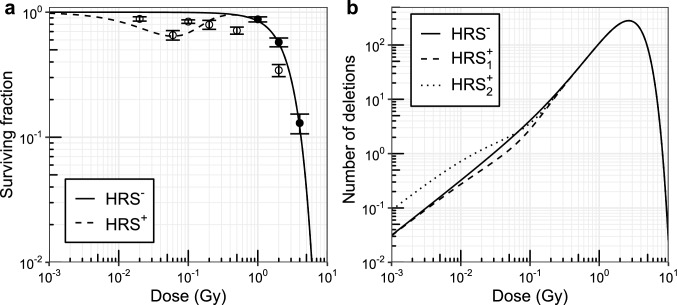
Effect of the cellular hyper-radiosensitivity (HRS) assumption on clonogenic cell survival and formation of the radiation-induced Sfpi1 deletion. **a** Cell survival was modeled under the assumption that target cells do (dashed curve) or do not (solid curve) exhibit HRS. The shown data represents the mean (±standard error) survival fractions of SLAM-HSCs (filled circles, *n* = 3, Mohrin et al. 2010) and long-term HSCs (open circles, *n* = 5) exhibiting HRS (Rodrigues-Moreira et al. 2017). **b** The number of cells with a radiation-induced Sfpi1 deletion surviving low-dose exposure decreases under the HRS$^+_1$ target cell assumption (HRS only affects cell survival, dashed curve) compared to the HRS$^-$ assumption (solid curve). In contrast, the assumption that HRS stimulates cell killing *and* the formation of the Sfpi1 deletion (HRS$^+_2$) results in more cells with Sfpi1 deletions after very low dose exposure (dotted curve) compared to the other assumptions

Figure 2a shows the surviving fraction for the HRS^−^ (solid) and HRS^+^ (dashed) target cell assumptions. The surviving fraction curves were obtained with a linear-quadratic model (HRS^−^ assumption) and an induced-repair model (HRS^+^ assumption, Eq. (11)). The model parameters (Table 1) were taken from Stouten et al. (2021) to describe the surviving fraction of gamma-irradiated Slam-HSCs (filled circles, Mohrin et al. 2010). Rodrigues-Moreira et al. (2017) showed that LT-HSCs exhibit low-dose HRS following in vitro exposure (open circles). Given this observation, the induced-repair model parameter $\alpha _\text {s}$ was chosen such that HRS was approximately maximized following exposure to a dose of 0.06 Gy, with a corresponding observed clonogenic surviving fraction of about 0.65 (open circles, Rodrigues-Moreira et al. 2017). Although the utilized induced-repair model parameters are unable to describe the available HRS data exactly, this does not have a large effect on the qualitative impact of HRS on the rAML incidence, which is the main focus of this study.

The radiation-induced deletion with Sfpi1 copy loss is responsible for the formation of pre-leukemic cells (Verbiest et al. 2015). Figure 2b illustrates the effect of the HRS$^-$ (solid curve), HRS$^+_1$ (dashed curve) and HRS$_2^+$ (dotted curve) assumptions on the formation of pre-leukemic intermediate cells. If HRS only affects cell survival (dashed curve), fewer pre-leukemic cells are formed at lower doses compared to what one would expect based on the conventional linear-quadratic model (solid curve). Whereas if HRS stimulates cell killing and the formation of the Sfpi1 deletion (dotted curve), more pre-leukemic cells are formed at lower doses. The three curves are equal for doses larger than about 0.3 Gy. Similar to Stouten et al. (2021), the maximum number of cells with an Sfpi1 deletion is formed after about 2.7 Gy of exposure. Further increasing the dose induces frequent cell death, hence explaining the observation that the number of cells with an Sfpi1 deletion approaches zero following exposure to higher doses.

### The rAML incidence is calculated with the diagnosis time distribution

**Fig. 3:**
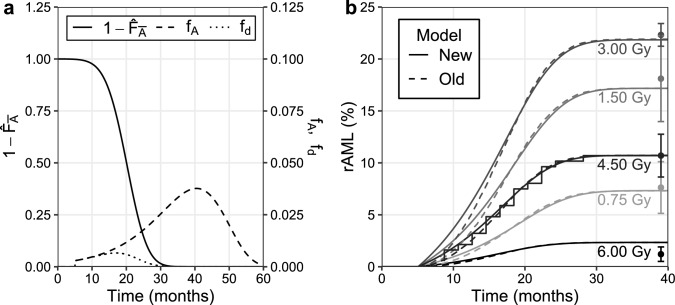
Quantification of time-dependent rAML onset. **a** The rAML diagnosis time distribution ($f_\text {d}$, dotted curve) is acquired by multiplying the potential rAML diagnosis time density, $f_\text {A}$ (dashed curve), with one minus the cumulative distribution function of deaths from non-rAML causes, 1-$\hat{F}_{\overline{\text {A}}}$ (solid curve). The area under the rAML diagnosis time curve represents the probability of developing rAML. **b** The time-dependent cumulative rAML incidence curves are shown following exposure to 0.75, 1.5, 3.0, 4.5 or 6.0 Gy (light gray to black) for the recently published model (dashed curves, Stouten et al. 2021) and the simplified model presented in this paper (solid curves). The cumulative incidence was determined by calculating the area under the diagnosis time curve $f_\text {d}$ as a function of time. The model was fitted to time-dependent cumulative incidence in CBA/H mice following 4.5 Gy of X-ray exposure (stairs) and the incidence data (mean ± standard error, *n* = 4,) shown at the end of the cumulative incidence curves (Major 1979; Mole et al. 1983)

The model presented in this paper was used to find the rAML diagnosis time distribution $f_\text {d}(t)$ (dotted curve, Fig. 3a). The distribution $f_\text {d}(t)$ (Eq. 1) was calculated after 4.5 Gy of exposure by multiplying the *potential* rAML diagnosis time distribution $f_\text {A}(t)$ (dashed curve), with one minus the cumulative distribution function for non-rAML death times $\hat{F}_{\overline{\text {A}}}(t)$ (solid curve). The probability of developing rAML was calculated by integrating the rAML diagnosis time curve $f_\text {d}(t)$.

Figure 3b shows the cumulative rAML incidence in time following exposure to doses of 0.75, 1.5, 3.0, 4.5 or 6.0 Gy (light gray to black). These curves were obtained by integrating the rAML diagnosis time distribution $f_\text {d}(t)$ as a function of time. The model presented here (solid curves) yields results similar to the previously published rAML model that is more complex and computationally intensive (dashed curves, Stouten et al. 2021). The initial rise in the cumulative rAML incidence proceeds faster with the new model compared to the previous version. The previous model contains an additional intermediate cell compartment such that cells with an Sfpi1 deletion do not have an initial growth advantage (Stouten et al. 2021), hence explaining the initial delay in rAML diagnoses observed with the previous model. Furthermore, as the previous model, the new model is also able to describe the total rAML incidence among male CBA/H mice (filled circles) and the time-dependent cumulative incidence data (stairs) (Major 1979; Mole et al. 1983).

### Low-dose HRS modifies the rAML dose-response curve

**Fig. 4:**
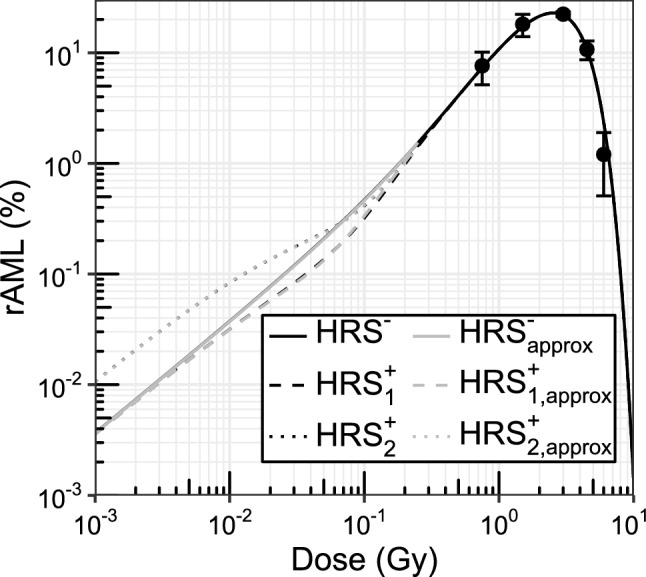
Hyper-radiosensitivity (HRS) modifies the rAML dose-response curve. The rAML dose-response curve obtained with the HRS^-^ assumption is linear-quadratic at lower doses (solid black curve). In the presence of HRS$^+_1$ target cells (HRS only affects cell survival), the low-dose incidence is reduced (dashed black curve) compared to the HRS^-^ assumption. The rAML incidence at very low doses is higher with the HRS$^+_2$ target cell assumption (HRS stimulates cell killing and the formation of the Sfpi1 deletion, dotted black curve). The modeled high-dose incidence estimates are in accordance with the available male CBA/H mouse data (Major 1979; Mole et al. 1983, standard errors are shown for *n* = 4). The modeled HRS$^-$ rAML dose-response curve (solid black curve) is almost identical to the linear-quadratic dose-response curve approximation *y*(*D*) = 3.63*D* + 10.1*D*^2^ made by Stouten et al. (2021) (HRS$^{-}_\text {approx}$, solid gray curve). The linear coefficient of the linear-quadratic response curve *y*(*D*) could be modified with simple dose-dependent expressions to approximate the dose-response curves obtained with the HRS$^+_1$ (dashed gray curve, Eq. 16) and HRS$^+_2$ (dotted gray curve, Eq. 17) assumptions

Figure 4 shows the modeled effect of low-dose HRS on the rAML incidence. The incidence curves were calculated by running the model for the previously discussed three HRS assumptions (HRS$^-$, HRS$^+_1$, HRS$^+_2$). First, similar to Stouten et al. (2021), the rAML incidence curve corresponding to the HRS^-^ assumption increases in a linear-quadratic manner with the absorbed radiation dose (solid black curve). Second, a comparison of the dose-response curves for the HRS$^-$ and HRS$^+_1$ (dashed black curve) scenarios indicates that HRS may reduce the rAML incidence with a maximum effect around 0.06 Gy. Third, the incidence curve obtained with the HRS$^+_2$ assumption (dotted black curve) has a relatively high slope at very low doses compared to the other HRS assumptions. The three modeled rAML incidence curves are identical at higher doses, regardless of the low-dose HRS assumption.

The high-dose model predictions are in line with available rAML incidence data of 0.75, 1.5, 3.0, 4.5 and 6.0 Gy X-ray irradiated male CBA/H mice (Major 1979; Mole et al. 1983). Maximum rAML induction is observed with the model after about 2.5 Gy of exposure, and the rAML incidence decreases with higher doses due to the depletion of pre-leukemic cells with an Sfpi1 deletion and increased mouse deaths from non-rAML causes.

Figure 4 additionally shows that the modeled HRS^−^ rAML incidence percentages up to 0.2 Gy (solid black curve) can be accurately approximated with the linear-quadratic dose-response curve, $y(D)= c_1D+c_2D^2$ (solid gray curve), with coefficients $c_1=3.63$ Gy^−1^ and $c_2=10.1$ Gy^−2^, obtained by Stouten et al. (2021) from modeled rAML incidence percentages. Coefficient $c_1$ of the linear-quadratic dose-response curve approximation can be modified to describe the HRS$^+_{1,2}$ scenarios:

16 $$\begin{aligned} c_{1,\text {HRS}^+_1}(D)&= c_\text {1,r} \bigg (1 - \Big (\frac{c_\text {1,s}}{c_\text {1,r}}-1\Big ) zD e^{-\frac{D}{D_\text {c}}} \bigg ), \end{aligned}$$

17 $$\begin{aligned} c_{1,\text {HRS}^+_2}(D)&= c_\text {1,r} \bigg (1 + \Big (\frac{c_\text {1,s}}{c_\text {1,r}}-1\Big ) e^{-\frac{D}{D_\text {c}}} \bigg ) , \end{aligned}$$

where the constant $z=1$ Gy^−1^ was introduced to make the product *zD* dimensionless. Note that the term for the HRS$^+_1$ assumption differs from the induced-repair model (Marples and Joiner 1993) due to the addition of a dose-dependency before Euler’s number; furthermore, the first plus sign was changed into a minus sign because this assumption is responsible for decreasing the rAML incidence. The term for the HRS$^+_2$ assumption is identical to the induced-repair model (Marples and Joiner 1993). Figure 4 illustrates that $c_{1,\text {HRS}^+_1}(D)$ (dashed gray curve, $c_\text {1,r}=c_1$ Gy^−1^, $c_\text {1,s} =71.9$ Gy^−1^, $D_\text {c}=0.06$ Gy^−1^) and $c_{1,\text {HRS}^+_2}(D)$ (dotted gray curve, $c_\text {1,r}=3$ Gy^−1^, $c_\text {1,s} =10.8$ Gy^−1^, $D_\text {c}= 0.026$ Gy^−1^) can be utilized to accurately approximate the modeled rAML incidence estimates corresponding to the two HRS assumptions (dashed and dotted black curves respectively).

Multiple observations can be made from $c_{1,\text {HRS}^+_1}(D)$ and $c_{1,\text {HRS}^+_2}(D)$. First, at very low doses, the slope parameter for the HRS$^-$ and HRS$^+_1$ assumptions are identical ($c_1=c_\text {1,r}=3.63$ Gy^−1^) and about three times smaller compared to the HRS$^+_2$ assumption ($c_{1,\text {s}}=10.8$ Gy^−1^). A relatively large slope parameter was expected for the HRS$^+_2$ scenario due to the assumption that low-dose HRS stimulates the formation of the Sfpi1 deletion. Second, as the dose increases, $c_{1,\text {HRS}^+_1}(D)$ becomes smaller compared to the HRS$^-$ scenario ($c_1$) due to the assumption that HRS only affects cell survival, which results in fewer Sfpi1 deletions and therefore lower rAML incidence. Third, if the dose becomes sufficiently large such that $D/D_\text {c}\approx 1$, terms $c_{1,\text {HRS}^+_1}(D)$ and $c_{1,\text {HRS}^+_2}(D)$ both approach the slope parameter $c_{1,\text {r}}$.

## Discussion

The surviving fraction curve of the cells possibly responsible for rAML development displays excess cell killing (HRS) at lower doses (Rodrigues-Moreira et al. 2017). The aim of the present study was to explore the possible effect of HRS on the rAML incidence in male CBA/H mice. Lower rAML incidence occurred in in-silico mice carrying HRS$^+_1$ target cells compared to HRS^−^ cells over the same dose interval for which hyper-radiosensitive surviving fractions were modeled. This incidence reduction arises because the probability of acquiring the Sfpi1 deletion decreases due to a sharp HRS-associated increase in cell killing. A lower number of viable pre-leukemic cells carrying the Sfpi1 deletion translates to a lower probability of malignant cell formation and rAML onset during a mouse’s lifespan, hence explaining the difference in rAML incidence depending on the HRS status.

Low-dose ionizing radiation exposure has been shown to increase the number of chromosomal aberrations and deletions (Seth et al. 2014; Troshina et al. 2020); therefore, a scenario was considered in which HRS stimulates cell killing and the formation of the Sfpi1 deletion. The rAML incidence at very low doses was increased for the HRS$^+_2$ target cell assumption compared to the other assumptions, since a higher number of pre-leukemic cells directly leads to increased rAML incidence. A scenario in which HRS only affects the Sfpi1 deletion was not considered because there are experimental indications that HRS affects cell killing (Rodrigues-Moreira et al. 2017). If HRS would only stimulate the Sfpi1 deletion, then the dose-response curve would still be similar to the proposed expression ($c_{1,\text {HRS}^+_2}(D)$, Eq. 17) at *very* low doses due to the stimulation of pre-leukemic cell formation. At higher doses, the effect of HRS disappears which causes the HRS$^+$ rAML dose-response curves to converge onto the HRS$^-$ curve.

Although low-dose HRS has been observed in vitro, 0.02 Gy irradiated C57BL/6-CD45.2 mice did not have significantly decreased LT-HSC counts compared to controls 4 months post-irradiation (Rodrigues-Moreira et al. 2017). This implies that low-dose HRS might not occur in vivo. However, a lack of significantly decreased cell counts may be attributed to: the presence of radiation-induced inactivated cells (i.e., cells that did not clonogenically survive irradiation, but are still present in the bone marrow), repopulation and/or a small low-dose-associated (0.02 Gy) effect size.

The induced-repair model from Marples and Joiner (1993) utilized in the present study and by Rodrigues-Moreira et al. (2017) may not be the appropriate model to describe low-dose HRS in LT-HSCs. The observed HRS-mediated decrease in cell survival is much steeper than what can be properly described through the induced-repair model. For example, with the reported parameters of $\alpha _\text {r}$ = 0.63 Gy^−1^ and $\alpha _\text {s}$ = 9.84 Gy^−1^ (Rodrigues-Moreira et al. 2017), the surviving fraction was reduced to 0.79 instead of the observed mean of about 0.65 following 0.06 Gy of exposure. Furthermore, the smaller radiosensitivity parameter $\alpha _\text {r}$ = 0.04 Gy^−1^ estimated by Stouten et al. (2021) was used here to describe cell survival and rAML, instead of the relatively large value found by Rodrigues-Moreira et al. (2017). A global optimization technique (simulated annealing) was employed in the present study to assess whether a good rAML model fit could have been obtained with $\alpha _r$ = 0.63 Gy^−1^. But this method failed to identify a realistic optimal parameter set capable of describing the rAML data. The presented rAML model requires a relatively small value of $\alpha _\text {r}$ to properly describe the upward curvature of the rAML incidence data up to about 2.5 Gy. Although the values of $\alpha _\text {r}$ and $\beta$ estimated by Stouten et al. (2021) can also be used to describe clonogenic survival data of HSCs and HSPCs (Mohrin et al. 2010), this finding might be a coincidence.

For the HRS$^+_1$ assumption, the quotient of the surviving fraction for the HRS$^-$ assumption divided by that of the HRS$^+$ assumption ($S/S_\text {HRS}$) was found to be identical to the quotient obtained for the number of radiation-induced pre-leukemic cells ($I_0/I_{0,\text {HRS}^+_1}$, results not shown). This was expected because the cells were assumed to die in accordance with the induced-repair model (Eq. 11), whereas the induction of the Sfpi1 deletion was described with the conventional linear-quadratic model. At lower doses, the rAML dose-response curve can be accurately described in terms of the number of radiation-induced pre-leukemic cells (Stouten et al. 2021), which implies that the Sfpi1 deletion is an important mutation that largely determines the shape of the dose-response curve (i.e., Fig. 2b explains Fig. 4). Therefore, the effect of the HRS$^+_1$ assumption on the rAML incidence was found to be approximately identical to the effect of HRS on cell survival and the induction of pre-leukemic cells. Although this observation follows from a model assumption, this could occur in vivo if the presented model is representative of the actual two-mutation major rAML disease pathway.

The mathematical mouse model was additionally redesigned such that the computation time is negligible, while yielding a similar linear-quadratic dose-response curve and time-dependent cumulative incidence curves compared to the more complicated and time-consuming rAML model developed by Stouten et al. (2021). Although most of the model assumptions and the linear-quadratic dose-response curve obtained with the rAML model were discussed in detail by Stouten et al. (2021), it is important to note that a different set of assumptions could yield a distinct dose-response curve. For example, certain dose-response curves reflecting hormesis or a threshold (Brenner et al. 2003) were excluded beforehand due to a lack of data on processes that might affect the response curve for the major rAML disease pathway.

Epidemiological studies have found that the dose-response curve for human AML risk can be described with a linear-quadratic model (Preston et al. 1994) and a preferred quadratic model (Richardson et al. 2009; Hsu et al. 2013). Similar to Stouten et al. (2021), the linear-quadratic dose-response curve obtained here was found through a bottom-up approach and should therefore not be extrapolated to humans due to differences in the underlying AML disease pathway (Verbiest et al. 2015). Most rAML cases in male CBA/H mice can be explained through the major rAML pathway involving the interstitial deletion with Sfpi1 copy loss and the Sfpi1 point mutation. The remaining cases occur through minor pathways that may be independent of the Sfpi1 deletion and/or the Sfpi1 point mutation (O’Brien et al. 2020). The overall rAML dose-response curve is the sum of the (different) dose-response curves corresponding to the major and minor rAML disease pathways (Stouten et al. 2021). It is possible that HRS can influence the expression of disease pathways in different ways since HRS has been observed for distinct endpoints, e.g., cell survival and mutations (Marples and Joiner 1993; Seth et al. 2014; Troshina et al. 2020).

It should be noted that HRS may affect cancer incidence in other ways. For example, Jacob et al. (2008) showed that, compared to the conventional linear-quadratic cell survival model, the incorporation of HRS in the two-stage clonal expansion model may increase the low-dose risk of mortality from all solid cancer types among male Japanese atomic bomb survivors. The obtained dose-response curve was similar to the HRS$^+_1$ rAML dose-response curve presented here, but instead of decreasing the incidence, HRS was found to increase the incidence. Higher cancer mortality risk was found by Jacob et al. (2008) due to the assumption that increased cell killing can temporarily increase the proliferation rate of intermediate cells to overcompensate radiation-induced cell inactivation. Ban and Kai (2009) made a similar observation regarding the effect of ionizing radiation on the proliferation rate. Based on the available data, Jacob et al. (2008) found that both the linear-quadratic and the induced-repair cell survival models could describe the available cancer risk data equally well. In the present paper, the assumption was made that ionizing radiation exposure does not influence the proliferation rate of pre-leukemic cells, hence the model-based observation that the rAML incidence is lowered if HRS only affects surviving cell fractions. Findings similar to Jacob et al. (2008) can be obtained with the presented model if a cell killing-dependent proliferation rate is assumed (results not shown).

Although cellular HRS has been thoroughly investigated (Lambin et al. 1993; Short et al. 1999; Joiner et al. 2001; Marples and Collis 2008; Olobatuyi et al. 2018), the available literature related to how HRS possibly affects low-dose cancer risk after acute exposure is scarce, which might be due to a lack of reliable biomarkers (Martin et al. 2013). The finding that low-dose HRS modifies the probability of rAML onset may not be limited to this form of cancer. In general, a consequence of HRS may be that this process changes the probability that certain radiation-induced (driver) mutations propagate/occur and contribute to long-term carcinogenesis. Then, acute doses absorbed during e.g. whole-body PET/CT scans may be sufficiently large to cause a small HRS-mediated increase/decrease in cancer risk compared to what is expected based on the linear no-threshold assumption. Two simple HRS terms were introduced here such that the linear coefficient of a risk model can be modified to include HRS. However, the application of these HRS terms should be limited to illustrative purposes because it requires parameters that cannot be identified from epidemiological data.

It may be possible to experimentally examine the presented hypotheses about the influence of HRS on the rAML incidence in male CBA/H mice. First, one should determine the dose-response curve for the number of cells with a deleted Sfpi1 copy. Data from Peng et al. (2009) indicate that bone marrow cells of mice irradiated with iron ions or X-rays may result in an HRS-dependent increase in Sfpi1 loss one day as well as 1 year after iron ion (CBA/Ca mice) and one month after X-ray (CBA/H mice) exposure. Unfortunately, insufficient data points are available to definitively confirm/reject the presented hypothesis about HRS with respect to the Sfpi1 deletion. Therefore, the experiment from Peng et al. (2009) should be repeated with more doses. Based on such an experiment, it is possible to examine the HRS$^+$ assumptions regarding the induction of the Sfpi1 deletion (Fig. 2b). Second, if HRS truly occurs in vivo during each irradiation event in male CBA/H mice, one could conduct a dose fractionation experiment to test whether HRS affects the rAML incidence. Consider total absorbed doses of 0.4, 0.8, 1.2, 1.6 and 2.0 Gy, delivered in 20 fractions over four weeks. A dose fraction may then fall within the HRS region e.g. 1.2/20= 0.06 Gy. For the HRS$^+_1$ assumption, one would expect to find fewer rAML cases for a dose fraction size that maximizes the HRS effect, whereas increased incidence could be detected if the HRS-mediated increased cell proliferation assumption from Jacob et al. (2008) is true. It should be noted that fractionated irradiation has been observed to induce repeatable HRS-mediated cell killing (Short et al. 2001; Turesson et al. 2010); however, whether repeatable HRS is induced depends on the cell line and interfraction time (Short et al. 2001). Therefore, it is vital to first test if HRS can be induced repeatably before conducting animal experiments.

Although a dose fractionation experiment was conducted by Mole and Major (1983), only four cases were observed following a total dose of 1.5 Gy (72 mice, 5.6%) and 3.0 Gy (65 mice, 6.2%) delivered in 20 fractions. No conclusions can be made about HRS based on these results, because they may have been obtained due to chance since the sample/effect size is too small given the large variation in rAML incidence that is usually observed within/between investigations with CBA/H mice (Major and Mole 1978; Mole et al. 1983; Mole and Major 1983; Olme et al. 2013b; Verbiest et al. 2018). The variation in incidence between earlier (Major and Mole 1978; Mole et al. 1983; Mole and Major 1983) and recent experiments (Olme et al. 2013b; Verbiest et al. 2018) may be attributed to a difference in housing conditions. Within an experiment, it may be easier to detect the possible effect of HRS on the rAML incidence by classifying each rAML case to the major or minor pathway based on the presence/absence of the Sfpi1 deletion.

## Conclusions

In conclusion, through a mathematical modeling approach it was shown how low-dose rAML incidence in male CBA/H mice may be influenced if HRS affects endpoints such as cell survival and the Sfpi1 deletion. For radiation protection, at the present state of knowledge, it is difficult to predict the relevance of HRS on cancer/leukemia incidence/mortality among humans. As discussed in the paper, HRS could either increase or decrease radiation-induced risk compared to what would be predicted by a linear or a linear-quadratic model. Through this work, a step has been set in the direction of expanding the limited available literature on the relationship between HRS and carcinogenesis. Furthermore, experiments have been proposed to identify the possible effect of HRS on rAML incidence, and investigate how the overall rAML dose-response curve can be described in terms of the minor/major rAML pathways.

## Data Availability

Data are available from the corresponding author upon request.

## Code availability

The R code required to reproduce all the results is available from the corresponding author upon request.
